# Ultrasound evaluation shows increase in laxity after partial common extensor origin detachment but not after additional lesion of the radial band of the lateral collateral ligament

**DOI:** 10.1007/s00167-021-06711-8

**Published:** 2021-08-28

**Authors:** Paolo Arrigoni, Davide Cucchi, Francesco Luceri, Andrea Zagarella, Michele Catapano, Alessandra Menon, Valentina Bruno, Mauro Gallazzi, Pietro Simone Randelli

**Affiliations:** 1 U.O.C. 1° Clinica Ortopedica, ASST Centro Specialistico Ortopedico Traumatologico Gaetano Pini-CTO, Piazza Cardinal Ferrari 1, 20122 Milan, Italy; 2 Laboratory of Applied Biomechanics, Department of Biomedical Sciences for Health, Università degli Studi di Milano, Via Mangiagalli 31, 20133 Milan, Italy; 3 Department of Biomedical Sciences for Health, Research Center for Adult and Pediatric Rheumatic Diseases (RECAP-RD), Università degli Studi di Milano, Via Mangiagalli 31, 20133 Milan, Italy; 4 Department of Orthopaedics and Trauma Surgery, Universitätsklinikum Bonn, Venusberg-Campus 1, 53127 Bonn, Germany; 5 U.O.C. Radiodiagnostica, ASST Centro Specialistico Ortopedico Traumatologico Gaetano Pini-CTO, Piazza Cardinal Ferrari 1, 20122 Milan, Italy; 6 Istituto Clinico San Siro, Via Monreale, 18, 20148 Milan, Italy

**Keywords:** Elbow, Ultrasound, Lateral collateral ligament, SMILE, Epicondylitis, Elbow tendinopathy, Tennis elbow

## Abstract

**Purpose:**

The lateral elbow musculature conveys a dynamic valgus moment to the elbow, increasing joint stability. Muscular or tendinous lesions to the anterior half of the common extensor origin (CEO) may provoke a deficiency in the elbow dynamic stabilizers, regardless of their traumatic, degenerative, or iatrogenic aetiology. Furthermore, a role for the radial band of the lateral collateral ligament (R-LCL) has been postulated in the aetiology of lateral elbow pain. This study aimed to evaluate the effects of sequential lateral releases with dynamic ultrasound, evaluating its capability to detect lesions of the CEO and of the R-LCL.

**Methods:**

Ultrasound investigation of the lateral compartment of the elbow was performed on nine cadaveric specimens with a 10 MHz linear probe in basal conditions, after the release of the anterior half of the CEO and after complete R-LCL release. The lateral joint line widening (*λ*) was the primary outcome parameter, measured as the linear distance between the humeral and radial articular surfaces.

**Results:**

The release of the anterior half of the CEO significantly increased *λ* by 200% compared to the starting position (*p* = 0.0008) and the previously loaded position (*p* = 0.0015). Conversely, further release of the R-LCL caused only a marginal, non-significant increase in *λ*.

**Conclusions:**

Ultrasound evaluation can detect changes related to tendon tears or muscular avulsions of the CEO and can depict lateral elbow compartmental patholaxity by assessing articular space widening while scanning under dynamic stress. However, it cannot reliably define if the R-LCL is injured. Iatrogenic damage to the CEO should be carefully avoided, since it causes a massive increase in compartmental laxity.

## Introduction

Both tendinous and ligamentous structures convey the lateral stability of the elbow: the collateral ligament (LCL) complex is a reinforcement of the lateral capsule, which consists of three components, including the ulnar band (U-LCL), the radial band (R-LCL) and the annular ligament [1–4]. Superficial to this structure, the wrist extensors contribute as dynamic stabilizers to lateral elbow stability. Among these, the extensor carpi radialis brevis (ECRB) gained popularity as possible source of lateral elbow pain, leading to the development of open and arthroscopic surgical release techniques to relieve pain. However, when examining the different approaches, conclusive evidence in favour of any technique is lacking; persistent post-operative pain is reported in a significant proportion of patients, and concerns about the possibility of an iatrogenic deficiency in the elbow dynamic stabilizers after ECRB release have been raised, questioning the role of ECRB release procedures [5–9]. The lateral elbow musculature conveys a dynamic valgus moment to the elbow, increasing joint stability and reproducibility of motion pathways by contraction [10–12].

The R-LCL is also believed to play a role in recalcitrant lateral elbow pain, but its pathology has not been extensively examined yet, as opposed to that of the U-LCL [13–16]. After a first description by Ciaudo et al. in 1980 [1] a possible role of this ligament in the aetiology of lateral elbow pain was postulated in the “symptomatic minor instability of the lateral elbow” (SMILE) concept [5]. However, as opposed to direct arthroscopic assessment, image-based preoperative diagnosis and classification of intra-articular findings and R-LCL abnormalities are challenging, and currently there is no gold standard in diagnosing lateral epicondylalgia [17, 18].

Ultrasound (US) examination is a first-step exam to approach lateral elbow pain, thanks to its low-cost and wide availability; it can detect changes related to tendon tears or muscular avulsions of the common extensor origin (CEO), evaluate U-LCL injuries, and depict compartmental patholaxity by assessing articular space widening while scanning under dynamic stress [19–22]. However, US sensibility in detecting lateral elbow instability has never been fully analysed and the available literature is limited to nonquantified and nonvalidated descriptions of US varus stress testing [20, 23–25]. Therefore, this study aimed to evaluate the effects of sequential lateral release with the dynamic US, evaluating the capability of this technique to detect tendinous tears at the level of the CEO and lesions of the R-LCL. The current study hypothesized that dynamic US evaluation of abnormal widening of the lateral joint line is sufficient to allow distinct identification of a simulated tear of the CEO and of the R-LCL. This will help define the role of US in the diagnostic approach to elbow instability and lateral elbow pain.

## Materials and methods

Institutional approval of the study protocol was obtained prior to study begin (Nicola’s Foundation & ICLO Research Center, ID19504).

Nine fresh-frozen cadaver specimens of upper extremities from human donors, including the complete middle third of the humerus and the entire hand, were available. Before the investigation, signs of previous trauma, stiffness, instability, or deformity were excluded. The distal radioulnar joint was transfixed with a 1.6 mm Kirschner wire in neutral position of the forearm to prevent undesired prosupination, since rotational movements are associated with muscular and ligamentous tension changes in the lateral aspect of the elbow [12, 26, 27]. Radiographs were taken to confirm integrity of bony structures, joint congruency, and correct Kirschner wire placement. The specimens were then mounted on a custom-made support designed to set elbow flexion and extension and allow controlled varus stress; the humeral position was set to mimic that obtained with the shoulder in 90° of forward flexion and 90° of internal rotation (i.e. lateral epicondyle facing upwards and transcondylar axis perpendicular to the floor).

Dynamic US examination of the lateral compartment was performed with a 10 MHz linear probe (Esaote MyLab 30, Esaote, Genova, Italy) placed on the skin of the lateral aspect of the elbow.

**Fig. 1:**
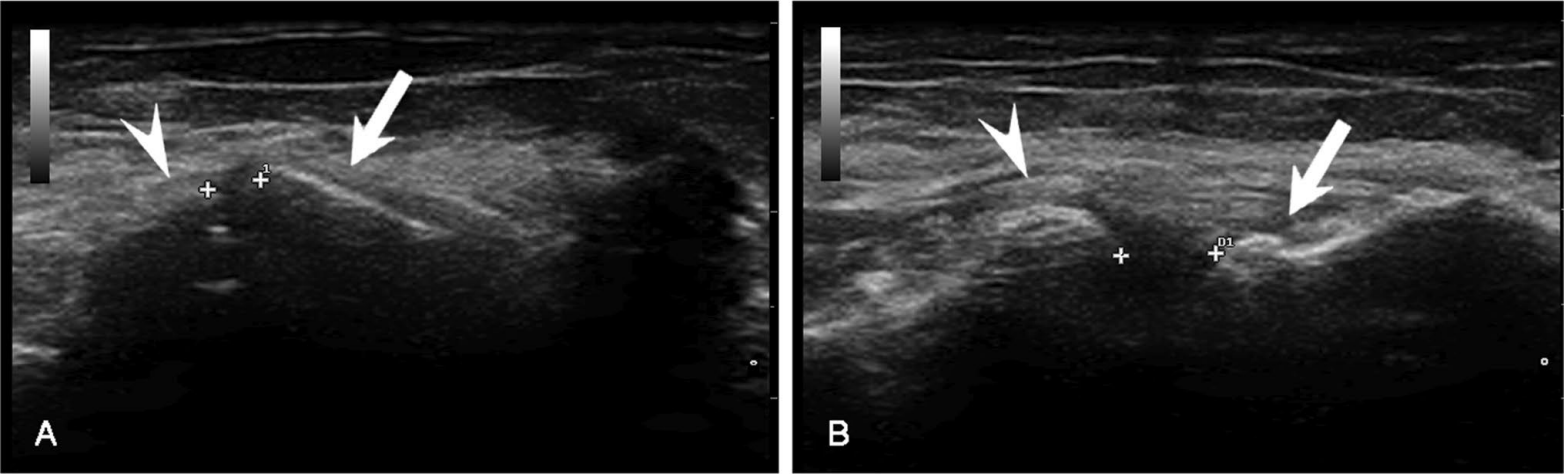
Dynamic ultrasound evaluation of the lateral compartment with 0.5 kg load applied to the hand before (**A**) and after (**B**) release of the anterior half of the common extensor origin. The arrow and the arrowhead indicate the lateral cortex of the radial head and the lateral cortex of the capitellum

To obtain standardized images with the most distinct bony articular margins, the transducer was oriented perpendicular to the radial head, centred in the coronal plane at the level of the midpoint of the radiocapitellar joint. The lateral joint line widening (*λ*), defined as the linear distance between the proximal end of the articular surface of the radial head and the articular surface of the capitellum, was considered as the primary outcome parameter and was measured in millimetres (mm) with electronic callipers with one decimal accuracy (Fig. 1).

Initial US investigation of the lateral compartment was performed in basal conditions, first with the elbow in full extension and then in 60° flexion and applied gravitational torque only. Subsequently, a 0.5 kg load was applied to the specimens’ hand, and the US evaluation was repeated in full extension and in 60° flexion.

A lateral approach according to Kocher was then performed on each specimen; the CEO was identified, and its midline was marked from the bony insertion to the myotendinous junction; the fibres were divided longitudinally along the midline, and the anterior half of the CEO was detached from the humeral epicondyle and reflected distally, exposing the underlying layer. Dynamic US investigation of the lateral compartment was repeated after this first soft tissue release (Fig. 1).

Finally, the R-LCL was identified and completely detached from its proximal insertion, and the US investigation was repeated.

All surgical procedures were performed by a single examiner with extensive experience in elbow surgery (P.A.). Care was taken to leave the anterior elbow joint capsule as intact as possible and not to damage the U-LCL, which lies under the unreleased posterior half of the CEO. Two dedicated musculoskeletal radiologists performed all dynamic US examinations, reaching mutual agreement on the obtained values (A.Z., M.C.).

### Statistical analysis

Statistical analysis was performed using GraphPad Prism v 6.0 software (GraphPad Software Inc.). Continuous variables were expressed as the mean ± standard deviation (SD) or medians and first and third quartiles [Q1–Q3], as appropriate. The Shapiro–Wilk normality test was used to evaluate the normal distribution of the sample and, if the null hypothesis of this test could not be rejected, the non-parametric Mann–Whitney test (*U* test) was applied for the analysis of the samples. Variables with a Gaussian distribution were analysed with Student’s *t* test. For all analyses, the significance level was set at a *p* value lower than 0.05. The sample size was based on similar publications dealing with releases and reconstructions of the lateral elbow ligaments; based on these previous reports, a number of *n* = 9 specimens was deemed appropriate to perform this study [28, 29].

## Results

Nine specimens underwent US evaluation in basal condition and after both releases (females: 66.7%; right elbows: 44.4%; mean age at death: 76.7 ± 15 years; transepicondylar axis: 6.2 ± 1.2 cm; forearm and wrist length: 29.6 ± 4.3 cm). No complications were encountered during the surgical procedures, and no difficulties emerged in performing US measurements. The capitellar and the radial articular surfaces were always easily identifiable, and *λ* could be calculated for each measurement.

### Effect of elbow flexion and gravitational torque

No significant differences were demonstrated when performing pairwise comparisons of the parameter *λ* measured in basal conditions with the elbow in full extension and in 60° of flexion, in both testing conditions with gravitational varus torque only and an additional 0.5 kg varus load. With the elbow kept in full extension, adding a 0.5 kg varus load did not produce significant changes in *λ* as compared to an extended elbow with gravitational varus torque only; on the other hand, with the elbow in 60° flexion adding a 0.5 kg varus load produced a minimal but statistically significant increase in *λ*, with an average elongation (Δ*λ*) of 46% (*p* = 0.0038, Table 1).

**Table 1:** Results of the initial ultrasound investigation before performing lateral releases

	Basal condition	0.5 kg varus stress	*p* value
Full extension	2.20 (±0.55)	2.63 (±0.81)	n.s
60° flexion	2.13 (±0.46)	3.11 (±0.45)	**0.004**
*p* value	n.s	n.s	

Bold indicates* p* < 0.05

The lateral joint line widening (*λ*) was measured as the linear distance in millimetres between the humeral and radial articular surfaces. Continuous variables were expressed as mean ± standard deviation (SD) or as median and interquartile range (first and third quartiles, Q1–Q3), as appropriate

*kg* kilogram, *n.s* not significant

Considering these results in the basal conditions, further analyses were performed with 0.5 kg varus load and with the elbow in 60° flexion, which is believed to better simulate the effect of varus loading on the lateral elbow in everyday activities [5].

### Effect of surgical release of lateral stabilizing structures

The surgical release of the anterior half of the CEO produced a mean increase in *λ* of approximately 3 mm, which corresponded to a statistically significant change in *λ* by + 200% as compared to the starting position (*p* = 0.0008).

**Fig. 2:**
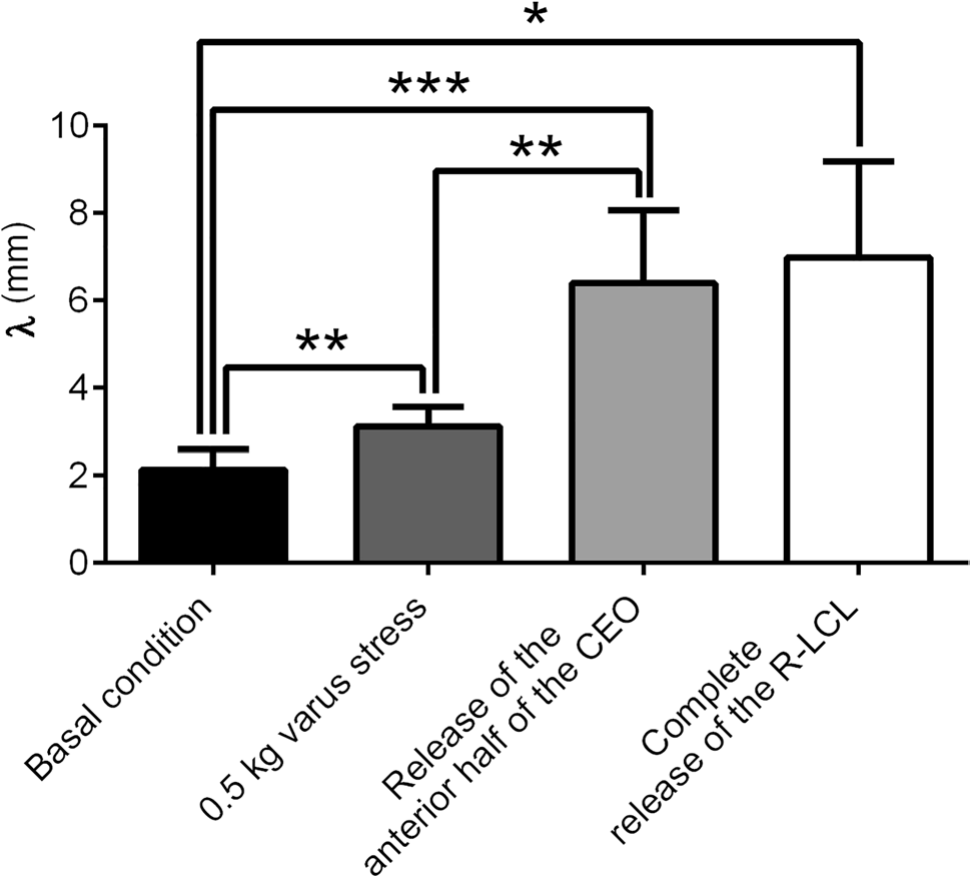
Effect of sequential lateral releases on the lateral joint line widening (*λ*). Only *P* values < 0.05 are indicated: *P < 0.05; **P < 0.01; ***P < 0.001. *CEO* common extensor origin; *kg* kilogram; ***λ*** lateral joint line widening; *R-LCL* radial band of the lateral collateral ligament

A further release of the R-LCL caused only a marginal increase in *λ* of less than 1 mm, which did not reach statistical significance compared to the previous release (Table 2, Fig. 2).

**Table 2:** Summary of the main study results: the lateral joint line widening (*λ*) was measured after sequential lateral releases

	Basal condition (no releases, no stress)	0.5 kg varus stress	Release of the anterior half of the CEO	Complete release of the R-LCL
	2.13 (± 0.46)	3.11 (± 0.45)	6.39 (± 1.67)	7.00 [5.25–7.65]
Elongation (% increase)	–	+ 46%	+ 200%	+ 228%
*p* value to basal condition		**0.004**	** < 0.001**	**0.016**
*p* value to previous release step			**0.002**	n.s

Bold indicates* p* < 0.05

Continuous variables were expressed as mean ± standard deviation (SD) or as median and interquartile range (first and third quartiles, Q1–Q3), as appropriate

*CEO* common extensor origin; *kg* kilogram; ***λ*** lateral joint line widening; *n.s* not significant; *R-LCL* radial band of the lateral collateral ligament

## Discussion

The most relevant finding of this study is that in a simulated setting of sequential soft tissue release of the lateral compartment of the elbow, US evaluation can detect changes related to tendon tears or muscular avulsions of the CEO but cannot reliably distinguish an associated R-LCL rupture. Dynamic US is, therefore, a suitable technique to evaluate compartmental laxity, which may be associated to traditional dynamic fluoroscopy; nevertheless, a second level imaging technique is necessary to provide a detailed assessment of capsular and ligamentous structures, which can neither be adequately visualized, nor provoke sufficient indirect changes.

Furthermore, this study showed that, in an outside-in release sequence, the peak increase in joint line widening (Δ*λ*) is obtained when releasing the anterior half of the CEO. Two relevant clinical consequences can be derived from this finding: first, iatrogenic damage to the CEO can produce undesired compartmental laxity, potentially leading to R-LCL overload and failure on the long run; second, intra-articular, arthroscopic R-LCL reconstruction procedures can be an option to address lateral elbow pain without damaging the CEO but require an intact overlying musculotendinous sheath to be successful.

Knowledge of lateral elbow anatomy is important to understand the pathology related to R-LCL abnormalities. However, the role of the R-LCL and of reconstructive procedures directed to this ligament is limited in literature, although its importance as lateral stabilizer has been suggested in biomechanical and clinical studies [1, 5, 26, 30, 31]. This broad diamond shaped ligament originates from the lateral epicondyle at a mean distance from its apex of approximately 7 mm and blends, in its distal part, with the fibres of the annular ligament, covering the broadest surface area among the lateral elbow ligaments [32, 33].

In the recently developed SMILE model, the role of the R-LCL as a static stabilizer was emphasized, suggesting that patholaxity and elongation of the R-LCL caused by mild, repetitive varus/pronation stresses can lead to relative hypermobility of the radial head, minor incongruence of the proximal radioulnar joint, subsequent intra-articular alterations and finally ECRB tendinopathy and lateral elbow pain [5]. This model is gaining popularity and an increasing number of reports are being published, reporting on MRI findings of R-LCL abnormalities in patients affected by recalcitrant lateral elbow pain [34–37].

Limited evidence exists regarding the specific contribution of the R-LCL to a pattern of minor elbow instability related to varus-pronation overload [38]. Previous studies mainly focussed on detecting major posterolateral rotatory instability in terms of pivot shift test, radial head translation or subluxation, or degrees of rotation or varus, being unable to detect a significant contribution of isolated sectioning of the R-LCL [26, 39–41].

These findings are only partially confirmed by the current study, which suggests that no US detectable significant changes in *λ* occur with sectioning the R-LCL after the previous muscular release; however, this study does not have the elements to define if the R-LCL gets loose before or after CEO damage. For surgeons approaching recalcitrant lateral elbow pain, this indicates that extra-articular open approaches releasing the CEO can produce an iatrogenic compartmental laxity, potentially leading to undesired R-LCL overload and failure. Further studies with a different setting are required to verify if reversing the release sequence (i.e. performing an intra-articular R-LCL release first) can lead to the appearance of US detectable changes and to define the possible role of arthroscopic approaches to address lateral elbow pain without damaging the CEO [42].

Preoperative diagnostic techniques to detect R-LCL patholaxity or elongation are still lacking. Some new clinical tests have been proposed to identify lateral pain of articular origin, but have not been validated yet on large cohorts [43]. Similarly, although US investigation has been demonstrated being to visualize the R-LCL effectively, only limited evidence exists on its capability to detect direct or indirect signs of R-LCL pathology [44, 45]. This study, which was designed to evaluate if dynamic US is capable of indirectly detecting a lesion of the R-LCL, distinguishing this from a tendon tear at the level of the CEO, could not confirm this hypothesis, suggesting that the *λ* is not a suitable parameter to evaluate this pattern of minor elbow instability.

This is probably caused by the fact that US indirect signs of lateral instability, including *λ*, are designed to investigate U-LCL lesions and no specific protocols are designed for the R-LCL [22, 46–48]. Therefore, efforts should be made to develop specific diagnostic protocols to better evaluate isolated R-LCL laxity, especially in patients affected by SMILE [36].

This study demonstrated that the US is suitable to indirectly evaluate integrity of the muscles and tendons of the lateral elbow, confirming the results of previous studies [20, 49, 50]. Muscles are important dynamic elbow stabilizers, as initially described by An et al. and Dunning et al. and more recently confirmed by Seiber et al. [10–12]. Among the extensor muscles, the ECRB plays a distinct role in lateral elbow stability: the position of its tendinous insertion, just extra-capsular and parallel to R-LCL, suggests a similar role of this muscle and the R-LCL [2]. Within the SMILE theory, the pathological elongation of the static stabilizer R-LCL could require the ECRB tendon to act as an extra-articular secondary dynamic stabilizer, resisting varus-pronation forces in support of a deficient or lax R-LCL, with tendinopathy being a possible consequence of excessive strain [5].

In this study, the release of the anterior half of the CEO, which includes the ECRB, significantly increased *λ* by 200% as compared to the starting position (*p* = 0.0008): this result recommends care when performing open surgical treatment of lateral elbow pathology or when repeating steroid injections in lateral epicondylitis, since an excessive release or an iatrogenic injury may trigger undesired instability [13, 36]; furthermore, it indicates that at the end of the a pathological cascade starting from patholaxity and elongation of R-LCL initially caused by mild, repetitive varus-pronation stresses, major stability changes may occur if the ECRB is involved. These findings support the hypothesis that R-LCL plication may work in reducing this instability pattern, direct reinforcing the R-LCL and thus unloading the ECRB [42]. Considering the results of this study, the authors advise against open approaches, which can lead to undesired damage to the CEO, with the risk of creating iatrogenic compartmental laxity.

The position of the forearm could also affect elbow stability. In the current study setting, the distal radioulnar joint was transfixed in neutral position to prevent undesired prosupination; the neutral position was chosen because it is the one which best resembles the position used in many office-based jobs, which is based on elbow suspension, varus stress and only minimal pronation of the forearm. However, conflicting results on the contribution of forearm rotation to elbow stability have been published [10, 12, 26, 27, 41, 51, 52], encouraging future studies aimed at comparing the differences in elbow laxity caused by R-LCL release at different degrees of forearm rotation. Flexion angles also affect ligamentous tension, but were not shown to affect the stability patterns significantly after muscular releases [12, 31, 41]. Our study included a preliminary analysis of the intact specimens with different flexion angles and different initial loads, which could not show any significant differences in *λ* between US examination with the elbow in full extension and in 60° flexion. Therefore, further analyses were performed only in 60° flexion and with 0.5 kg varus stress, which is believed to better simulate the effect of varus stress on the lateral elbow in everyday activities.

Limitations of this study include that it is an anatomical study on a limited number of specimens, examined in an open setting, which allowed performing soft tissue release only in a single sequence. Like the arthroscopic one proposed by McAdams et al. a different setting, could be helpful for future studies to verify if reversing the release sequence produces similar US changes [40]. Unfortunately, the subjective evaluation of the results chosen by McAdams and colleagues did not allow comparison with the results of the current study. Future studies should also consider a larger sample size, since the chosen one was based on previous publications dealing with releases and reconstructions of the lateral elbow ligaments and not on a dedicated power analysis [28, 29]. The contribution of progressive tissue creep on the laxity measurements was not evaluated; however, adequate preconditioning of the specimens was performed, cyclic loading was avoided, and care was taken to maintain the specimen moisture and temperature at constant levels [53]. Nevertheless, the authors suggest care when transferring these results to clinical practise, since the biomechanical properties of fresh-frozen cadaveric specimens may differ from that of living tissue.

Finally, the US is considered an operator-dependent investigation: to reduce bias, two dedicated musculoskeletal radiologists were involved for the whole duration of the study and performed all US examination together, reaching mutual agreement on the obtained values. Magnetic resonance imaging is regarded as a second level diagnostic tool established as gold standard for detecting capsular and ligament lesions [36]. However, it is expensive, time consuming, and was not available for a biomechanical cadaveric study involving repetitive evaluations and stress testing [34, 35, 37]. Furthermore, magnetic resonance imaging lacks functional and dynamic information, which is an advantage of US techniques.

## Conclusion

US evaluation of the lateral compartment of the elbow can detect changes related to tendon tears or muscular avulsions of the common extensor origin, and it can depict compartmental patholaxity by assessing articular space widening while scanning under dynamic stress. However, it cannot reliably define if the R-LCL is injured. The increase in *λ* obtained when releasing the anterior half of the CEO suggests care should be taken when performing open surgical treatment of lateral elbow pathology or when repeating steroid injections in recalcitrant lateral elbow pain, since iatrogenic undesired compartmental laxity could lead to R-LCL overload and failure, with subsequent aggravation of intra-articular pathologies.
